# Antecedents of behavioral intentions for purchasing safety tools among women

**DOI:** 10.1016/j.heliyon.2023.e17994

**Published:** 2023-07-05

**Authors:** Ardvin Kester S. Ong, Tyrone Wyeth O. Arceno, Allyza R. Padagdag, Wayne Ralph Lee B. Saragat, Hershey Reina Mae S. Zuñiga, Ma Janice J. Gumasing

**Affiliations:** a School of Industrial Engineering and Engineering Management, Mapúa University, 658 Muralla St., Intramuros, Manila, 1002, Philippines; b E.T. Yuchengco School of Business, Mapua University, 1191 Pablo Ocampo Sr. Ext, Makati, Metro Manila, 1205, Philippines; c Young Innovators Research Center, Mapúa University, 658 Muralla St., Intramuros, Manila, 1002, Philippines

**Keywords:** Purchasing intentions, Safety, Safety tools, Violence, Women

## Abstract

The rise of crimes and violence against women are evident worldwide. The self-protection and behavioral aspects however are underexplored. The need for assessment for safety such as the purchasing intentions of safety tools should be considered to promote the mitigation and reduction of violence and crimes against women, may it be in households or outdoors. This study aimed to holistically analyze the determinants of behavioral intentions to purchase self-defense tools among women. Focusing on the Philippines, the structural equation modeling considering the extended theory of planned behavior (TPB) and protection motivation theory (PMT) integrated model was completely analyzed. A total of 553 valid respondents were purposively gathered to completely assess the behavioral domains and protective behaviors of women. From the results, self-efficacy showed the most significant factor affecting purchasing intentions, followed by behavioral intentions, and the behavioral domains under TPB. Moreover, indirect effects of PMT variables to purchasing intentions were seen; highest from understanding of safety, perceived risk, and then perceived severity. The extended latent variable such as perceived safety showed an indirect effect while buying impulse showed a direct significant effect on purchasing intentions. This is considered as the first study that assessed the different variables affecting purchasing intentions of self-defense tools. The findings of this study would provide a gateway as benchmark for how women feel, behave, and seeks protection. Implementing the suggestion with other aspects would end up promoting the overall safety of a country, not just for women, but for all victims against crime and violence.

## 1 Introduction

Safety preparation and practices have been said to be prominent in women. Dealing on consumer behaviors, women are generally implicated to be more active when products are focused on health-protective, safety, and risk-related purchasing intentions and behaviors [1]. Logan and Walker [2] have indicated the focus on women when topics revolve around safety, crime, victimization, and threats. Their discussion has presented how women are more vulnerable which is why they are common targets when it comes to risks. When it comes to this victimization, the study of Brown et al. [3] explained that demographic factors such as the educational attainment, age, and gender plays a significant mediating role – highlighting how females have more generalized threat as compared to male.

Reports from the World Health Organization [4] presented that a 736 million estimate of women are victims of different crimes. This shows that one out of three women are victims of physical, sexual, or both abuses – with age ranging from 15 years old and above. It was also added that a global count of 81 thousand women in general have been killed in 2020 – mostly violent even at the hands of their own family or partner. UN Women [5] showed that Asian countries have seen an increase in online search for help during the COVID-19 pandemic. With that, the problem of violence against women are continuously developing which needs to be explored.

One Asian country, the Philippines, have been stated to be higher than the United States in terms of violent crimes (28% more in murder rate per millions) [6]. Caliwan [7] presented a decrease in the late 2022 of crime rates in the country (6.37%), 105,568 incidents from 112,746 of 2021 in the Luzon region. However, it was also reported that other regions in the Philippines such as Visayas increased by 5.65%. Only a 1.27% increase in crime rate solution efficiency was seen throughout the year. Statista Research Department [8] presented a breakdown of crimes in the country per region, during the early periods of 2022. National Capital Region (NCR), the capital of the country, was reported to have 7330 crimes against the second highest with 4104 in region 4 at Visayas. It could be deduced from the different reports that the need for self-defense tools is needed in order to help protect oneself, especially among women who are more vulnerable to crimes and violence [9]. However, limited to no studies were seen in relation to self-protecting behavior among women.

Scarce literature regarding self-protection and self-defense tools, especially in the field of consumer behavior has been seen. Runyan et al. [10] focused on US women strategic choices for self-defense tools from violence. They considered women from 18 years old and above, which indicated that they are hesitant to do things they want due to fear of violence. Most of which considered to carry guns, weapons, devices, and strategies at home for self-protection. Moreover, their study suggested to consider other country context for self-defense choice behavior. Mirza and Sajid [11] created a framework encompassing pre-emptive self-defense and law regulations. Pierre [12] focused on the psychological factors affecting the use of guns as self-defense tools in America. They expounded on the different factors which encompasses behavioral aspects for the use of guns, including fear, risk perception, motivated reasoning on self-defense, attitude, culture, and cognitive aspects. Consensus was suggested to be considered, demographic diversity, and more research to encompass a more diverse finding. Chavan et al. [13] considered women in Mangalore regarding practices on self-defense. Their study focused on sexual harassment found that knowledge and understanding regarding the violence is a contributing factor relating to the use of self-defense tools. Strybel and Kumar [14] considered pepper spray for civilian use as self-defense tool. Their study focused on the actual use of the tool such as response time, physical use, and ability to use. Lastly, O'Meara [15] considered self-defense for imminent armed attacks. Their study mostly focused on the law relationship of self-defense on international law. With the scarce literature, the need to promote the use and behavioral intentions for self-defense tools is needed to be explored. Especially among women, the availability and marketability of self-defense tool would help reduce their victimizations.

In order for the holistic assessment of the factors affecting purchasing intentions of self-defense tools, different frameworks may be considered such as the theory of planned behavior (TPB) and protection motivation theory (PMT). TPB as explained in the study of German et al. [16] is a framework that completely assess the behavioral aspects of an individual. On the other hand, Author 2 et al. [17] expounded on the PMT framework as a means to assess the stress and coping appraisals of an individual. To which, both these theories have been considered and integrated to completely assess the behavioral aspects of stress and coping for safety reasons [18,19]. Though several literatures have extended the TPB in different context, the analysis of safety from self-defense tools have mostly covered interviews and multi-sectional design [[10], [11], [12], [13], [14], [15]].

This study aimed to holistically analyze the determinants of behavioral intentions to purchase self-defense tools among women. Focusing on the Philippines, the structural equation modeling (SEM) considering the extended TPB and PMT integrated model was completely analyze. This is considered as the first study that assessed the different variables affecting purchasing intentions of self-defense tools. Contributing to the literatures, the findings of this study may serve as a basis for theoretical foundation, even marketing strategies and promotion for self-defense tools. The results may be applied for promotion and advertisement of self-defense tools among women in developing countries. Moreover, this study was also able to provide implications on the behavioral, managerial, and protective aspects among women.

## 2 Conceptual framework

**Fig. 1:**
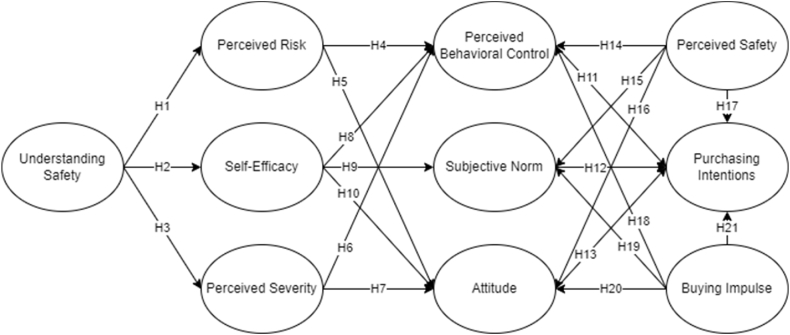
Conceptual Framework of the study.

This study considered both the extended TPB with PMT for holistic measurement of purchase intention of safety tools. Fig. 1 represents the conceptual framework utilized in this study with 21 hypotheses; 3 of which are under PMT, 7 connecting both theories, and 11 for the extended TPB latent variables. The presentation of the creation of hypotheses to support the integration are presented in this section, showing all the emerging interrelationship of latent variables.

Under the PMT, understanding is one of the core exogenous latent variables which correlated to the different threat and coping appraisals of an individual [17]. The study of Osca and Lopez-Araujo [20] showed how great understanding can reduce risks and accidents among women. It was seen from their study that separation of gender among analysis should be provided and not simply as a control of respondents. The difference in gender presents contrasting results which is why the current study focused on women. The study of Larrieta-Rubin de Celis [21] showed that great understanding among women to reduce violence, even in workplaces, should be employed to reduce their risk, severity of aftermath, and promote their self-efficacy. In addition, Martin-Fernandez et al. [22] also showed how vulnerable working women are, affecting work and family as well, and causes accidents. These results showed that vulnerability and effect of violence among women are evident. The risk promotes self-efficacy and heightens the perception of severe violence. Understanding of safety therefore plays a huge role for women's perception. However, the study of Seibokaite et al. [23] presented no effectivity of training safety among women but are positive among men. They expounded on the different attributes based on gender – suggestion different programs for training hazards, tailored on different genders. Thus, this study presented the different hypotheses.

H1 Understanding safety has a direct significant effect on perceived risk.

H2 Understanding safety has a direct significant effect on self-efficacy.

H3 Understanding safety has a direct significant effect on perceived severity.

For the different correlation of PMT and TPB, several studies have shown how the coping and threat appraisals affected people's behavioral aspects. Hou and Wu [24] showed that women's perceived risk and severity has promoted a positive behavioral response when it comes to rescue activities, decision making, and being a breadwinner. The attributes of behavioral domains are correlated with the different stress and coping appraisals due to their prominent gender equity engagement. In another study by Arnold et al. [25] the perceived risk and perceived severity among women have related to their desire and motivation to perform a positive or negative act. In the case of their study, when danger is perceived to be high, a likely escape was evident. In relation to this study, we wanted to highlight the correlation of perceived risk, perceived severity, and self-efficacy for self-protection by purchasing self-defense tools. Since gender does play a significant role among daily lives of individuals, a rehash on the promotion of reduced risk is needed [26]. Even in workplaces, Fontaneda et al. [27] showed the differences among gender. Women were more likely to be involve in risks and accidents and showed that they are more vulnerable when travelling or outside than in the usual workplace. The ability for them to have control over their behaviors, attitude, and effect by others are positive due to the circumstances presented.

For the integrated TPB and PMT theories, most studies have considered this in natural disasters such as that of German et al. [28]. They focused on response to preparedness when volcanic eruption strikes. A high relationship was seen among the domains of both theories which justifies their relationship. Similarly, Author et al. [29] showed the relationship when it comes to preparedness before earthquakes. Their study presented that people's perception of risks and severity affected their behavioral attributes. Lastly, Kurata et al. [18] showed that people's relatedness to the aftermath and effects of natural disasters promoted positive behaviors to help and mitigate disaster response. No studies have evaluated the domains of both theories in the field of safety in terms of self-protection. Llorens et al. [30] explained that a positive relationship is seen among behavioral attributes in women if the environment is seen to be safe, and otherwise. Therefore, the following were hypothesized.

H4 Perceived risk has a direct significant effect on perceived behavioral control.

H5 Perceived risk has a direct significant effect on attitude.

H6 Perceived severity has a direct significant effect on perceived behavioral control.

H7 Perceived severity has a direct significant effect on attitude.

H8 Self-efficacy has a direct significant effect on perceived behavioral control.

H9 Self-efficacy has a direct significant effect on subjective norm.

H10 Self-efficacy has a direct significant effect on attitude.

Among studies considering TPB, the domains are always affecting the intentions of individuals – either positively or negatively. For example, Islam et al. [31] showed different studies through an extensive literature review among purchasing intentions using TPB. The three domains were explained to be predominantly affecting the positive or negative purchasing intentions depending on people's need. Kumar and Nayak [32] performed a meta-analysis for pair-wise comparison among correlated attributes under the TPB domains for intention. It was seen that attitude was the most affecting variable towards the behavioral intention for purchasing, followed by subjective norm, and perceived behavioral control. Other factors were indicated to be affecting people's intention such as culture and demographic characteristics. On the other hand, the study of Liu et al. [33] showed that perceived behavioral control greatly affected the purchasing intentions when green products are considered, followed by attitude, and subjective norm. The differences in degree of purchasing intentions are dependent on the product itself, and therefore cannot be generalized. For future events and self-protection, Lou et al. [34] showed that the control of one's behavior is prominent. In terms of after COVID-19 lockdown, subjective norm was seen to be one of the most contributing factors affecting purchasing intentions [35]. In protecting intentions, Kurata et al. [18] showed that attitude was the dominant variable among the three domains. To which, three TPB domains were hypothesized to be.

H11 Perceived Behavioral Control has a direct significant effect on purchasing intentions.

H12 Subjective norm has a direct significant effect on purchasing intentions.

H13 Attitude has a direct significant effect on purchasing intentions.

The study of Weyrich et al. [36] showed that protective behaviors among people would be dependent on the protective measures that they utilize. In relation to this study, the perception of safety would affect their behavioral intentions to protect themselves, such as purchasing safety tools. Author 2 et al. [17] also showcased the effect of perceived safety to the behavioral intentions and attributes of individuals – especially from the household perspective. When it comes to necessity of protective behavior, people would be less likely to take precautionary measures if they perceived the environment to be relatively safe. However, the discussion of Caliwan [7] showed that the country considered in this study, whether rural or urban, are prominent to crimes and accidents. In relation, the behavioral domains are seen to be affected whether positively or otherwise if risk is prominent which would affect a person's health [37] or well-being [38]. Thus, it was hypothesized that.

H14 Perceived safety has a direct significant effect on perceived behavioral control.

H15 Perceived safety has a direct significant effect on subjective norm.

H16 Perceived safety has a direct significant effect on attitude.

H17 Perceived safety has a direct significant effect on purchasing intentions.

Individuals who purchase an item for any reason may be affected by impulse. As explained by Liu et al. [35], stimulus such as an event may cause a positive behavioral intention for purchasing. As presented in their study, the stimulus-organ-response was expanded with TPB to showcase buying behavior. Their results indicated a positive response on purchasing consumer behavior when stimulus cues are present. Trivedi et al. [39] highlighted that women are more impacted to have buying impulses which affects their behavior and purchasing intentions. Similarly, Um et al. [40] presented buying impulse to be ubiquitous among consumers – which affects their behavior attributes. Wang and Li [41] explained that when it comes to safety, positive behavioral attributes are evident for buying and purchasing intentions. The study related impulse buying through hedonic motivation, showing that people's purchasing behavior is relative due to their perception. In relation to this study, buying impulse due to an increase in risk, violence, and danger is evident in the current generation may affect their behavior and purchasing activities. Therefore, the following hypotheses were created.

H18 Buying impulse has a direct significant effect on perceived behavioral control.

H19 Buying impulse has a direct significant effect on subjective norm.

H20 Buying impulse has a direct significant effect on attitude.

H21 Buying impulse has a direct significant effect on purchasing intentions.

## 3 Methodology

### 3.1 Participants

The current study considered purposive sampling through an online survey which was distributed through different social media platforms and groups. A total of 553 valid responses was obtained as presented in Table 1. Only female respondents who have the capability to buy self-defense tools were considered in this study. As explained in the study of Author 2 et al. [17], a minimum of 250 respondents would be needed to have a good structural equation model. In addition, German et al. [16] explained that 400 valid respondents would be needed to generalize 62.6 million Filipinos with 95% significance level. This study surpassed both conditions and could be a representation of Filipino women for purchasing intentions of safety tools.

**Table 1:** Descriptive statistics of the respondents (n = 553).

Characteristics	Category	N	%
**Age**	Less than 18 years old	23	4.20
	18–25 years old	265	47.9
	26–30 years old	198	35.8
	31–38 years old	15	2.70
	39–45 years old	17	3.10
	46–50 years old	28	5.10
	51 years old and older	7	1.30
**Average Travel Time**	Less than 2 h	21	3.80
	2–4 h	344	62.2
	4–6 h	176	31.8
	6–8 h	7	1.30
	More than 8 h	5	0.90
**Average monthly income/allowance**	Less than 15,000 PhP	430	77.8
	15,000–30,000 PhP	64	11.6
	30,001–45,000 PhP	24	4.30
	45,001–60,000 PhP	13	2.40
	60,001–75,000 PhP	4	0.70
	More than 75,000 PhP	18	3.30
**Residence**	Rural	129	23.3
	Urban	424	76.7
**Do you have life insurance?**	YES	175	31.6
	NO	378	68.4
**Which among the safety tools would you consider?**	Pepper Spray/Mace	167	30.2
	Pocketknives	53	9.53
	Stun Guns/TASER	158	28.6
	Tactical Knives/Pens	24	4.34
	Alarms	132	23.9
	Spikes	19	3.43

From Table 1, the descriptive statistics among the demographics presented that majority are within 18–25 years old (47.9%) and 26–30 years old (35.8%), while the rest are older. Following which is the average travel time (daily), majority with 2–4 h (62.2%) and 4–6 h (31.8%). As explained in the transportation study of Cahigas et al. [42], most Filipino utilize the public utility vehicles for travelling to work or school – which is this study's demographics. At the same time, the Philippine travel advisory [43] had advised people to be cautious when travelling to different destination may it be near or far due to the increase in crime rates. Thus, it could be deduced that a representation for this study has been achieved. On the other hand, most of the respondents have lower monthly allowance or income (77.8% and 11.6%), living in urban residence (76.7%). This study tried to equalize the demographic statistics but obtained only 23.3% from the rural residences. Presented by Caliwan [7], crime rates are present in different regions of the Philippines. Most of which are from the capital of the country, and some are from rural areas, thus no issue will be present from the collected data. Lastly, most of the respondents do not have any life insurance (68.4%) compared to those who have (31.6%). Which shows the need for self-protection.

### 3.2 Questionnaire

Presented in appendix section are the items utilized in this study. Obtained from related studies presented in section 2, 40 adapted questionnaire items were considered. To which, 16 items are for the PMT variables, 12 for TPB, 8 for the extended buying impulse and perceived safety variables, and 4 for purchasing intentions (Supplementary Materials). Considering a 5-point Likert Scale following Cahigas et al. [42] and Author 2 et al. [17], complete answers from all respondents were obtained. In addition, this study was approved by Mapua University Research Ethics Committees (FM-RC-23-01-01). Moreover, Informed consent was obtained from all subjects involved in this study (FM-RC-23-02-01).

### 3.3 Structural equation modeling

Different fields involving consumer behavior, when analyzed using frameworks such as TPB and/or PMT provides nonlinear relationship. To which, mediators are available which may affect the analysis wherein, structural equation modeling (SEM) is utilized to determine the causal effects among latent variables [44]. For the study of Islam et al. [31], determination of factors affecting behavioral intentions to buy apartments utilized the SEM analysis. Dash and Paul [44] expounded on the difference among types and software for SEM analysis. PLS-SEM using SMART PLS and CB-SEM using AMOS have classifications for the analysis. For lower sampled data, PLS-SEM is recommended. However, if the response rate is higher, AMOS may be utilized. In addition, established frameworks are suggested to be analyzed with CB-SEM and PLS-SEM otherwise. With this study, both the individual theories and its integration have been established – to which, CB-SEM using AMOS was considered. Similarly, Liu et al. [33] utilized SEM to assess intention to choose for a green product purchase among consumer using the extended TPB. With that, this study considered AMOS 25 for the CB-SEM analysis of determinants affecting purchasing intentions of safety tools.

## 4 Results

**Fig. 2:**
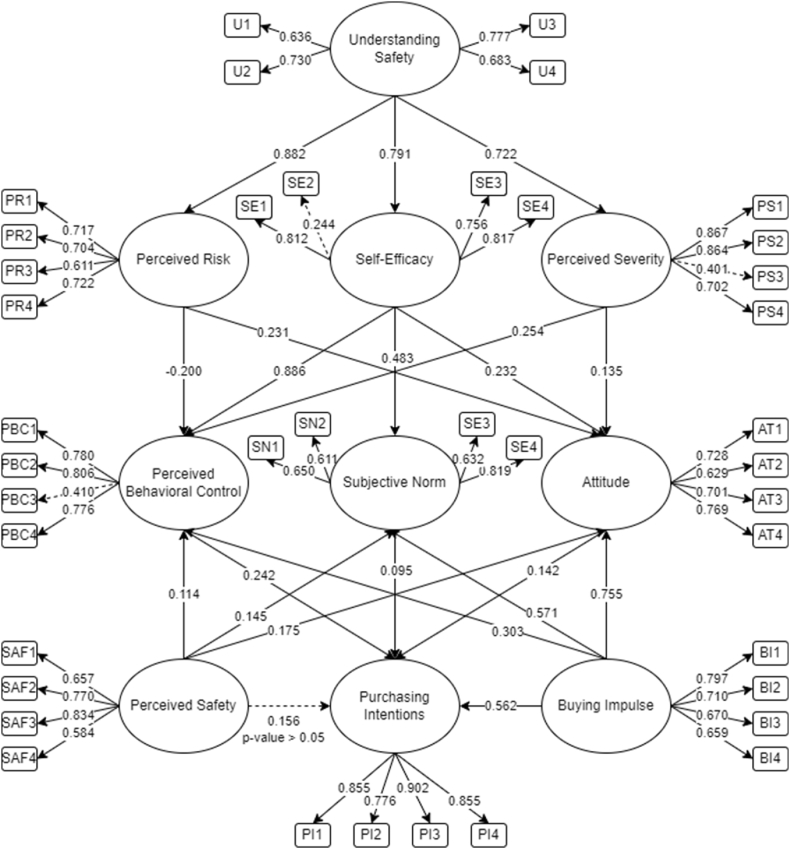
Initial SEM with indicators for purchasing intentions of safety tools.

The initial SEM for purchasing intentions of safety tools is presented in Fig. 2. From the initial findings, only perceived safety on its effect with purchasing intention was deemed insignificant (hypothesis 17). Following the suggestion of Hair [45], p-value greater than 0.05 is considered insignificant and should be removed. Aligned with the discussion, those items with less than 0.50 values are also insignificant and should be removed [45]. Thus, SE2, PS3, and PBC3 were removed. Utilizing AMOS 25, the final SEM run was conducted after the removal of insignificant relationship and items.

**Fig. 3:**
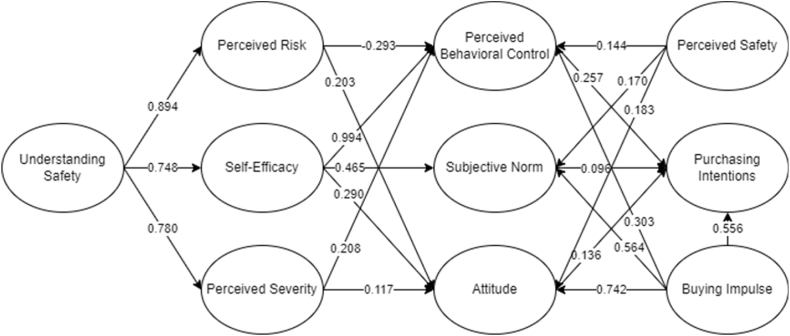
Final SEM for behavioral intentions to purchase safety tools.

Presented in Fig. 3 is the final SEM for the purchasing of safety tools among women. It could be seen that 20 out of 21 hypotheses were considered significant and are accepted. For the measured items, the descriptive statistics are presented in Table 2. There is a total of 37 out of 40 items that were deemed significant and could be utilized to measure the different unobserved latent variables [44].

**Table 2:** Descriptive statistics of measured items.

				Factor Loading	
Factor	Items	Mean	Std. Deviation	Initial	Final
	U1	4.2242	0.97076	0.636	0.639
Understanding Safety	U2	4.6293	0.69026	0.730	0.726
	U3	4.1989	0.99284	0.777	0.779
	U4	4.5570	0.77138	0.683	0.680
	PR1	4.0416	1.00366	0.717	0.724
	PR2	4.3635	0.87636	0.704	0.712
Perceived Risk	PR3	3.4792	1.16097	0.611	0.662
	PR4	4.6926	0.67531	0.722	0.739
	SE1	3.7848	1.14160	0.812	0.848
Self-Efficacy	SE2	3.0108	1.09506	0.244	–
	SE3	4.2405	0.90591	0.756	0.746
	SE4	3.2550	1.13016	0.817	0.859
	PS1	4.6546	0.65252	0.867	0.875
Perceived Severity	PS2	4.6510	0.67786	0.864	0.876
	PS3	4.4521	0.85654	0.401	–
	PS4	4.5732	0.75095	0.702	0.683
Perceived Behavioral Control	PBC1	4.4846	0.76627	0.780	0.788
	PBC2	3.7342	1.03378	0.806	0.810
	PBC3	4.5063	0.76876	0.410	–
	PBC4	4.3526	0.82778	0.776	0.767
	SN1	4.1591	0.97430	0.650	0.685
Subjective Norm	SN2	4.1049	1.01788	0.611	0.645
	SN3	3.5533	1.23848	0.632	0.667
	SN4	3.0778	1.11776	0.819	0.820
Attitude	AT1	4.3580	0.86927	0.728	0.731
	AT2	3.8590	1.11072	0.629	0.627
	AT3	4.3671	0.91928	0.701	0.797
	AT4	3.8969	1.18193	0.769	0.766
	PS1	3.4702	1.17935	0.657	0.658
Perceived Safety	PS2	3.2640	1.19438	0.770	0.770
	PS3	2.8626	1.28646	0.834	0.834
	PS4	3.9584	1.10830	0.584	0.584
	BI1	3.9114	1.10717	0.797	0.797
Buying Impulse	BI2	4.1736	1.00662	0.710	0.715
	BI3	4.0325	1.04206	0.670	0.669
	BI4	4.1067	1.02567	0.659	0.662
Purchasing Intentions	PI1	3.8626	1.09309	0.855	0.855
	PI2	3.1646	1.20538	0.776	0.775
	PI3	4.1248	1.00757	0.902	0.902
	PI4	3.2731	1.30895	0.855	0.854

In addition to the different threshold for acceptable items, analysis of the overall measure was conducted using Cronbach's alpha with threshold 0.70, together with the composite reliability. In addition, the average variance extracted was calculated with a threshold of 0.5. Anything below was suggested to be acceptable as long as the composite reliability is high [45]. From the findings, all items are within the set threshold as presented in Table 3. From the results, all items surpassed the suggested threshold and are valid for analysis and interpretation [44,45].

**Table 3:** Composite reliability and validity.

Factor	Cronbach's α	Composite Reliability (CR)	Average Variance Extracted (AVE)
Understanding Safety	0.755	0.800	0.501
Perceived Risk	0.725	0.802	0.504
Self-Efficacy	0.795	0.859	0.671
Perceived Severity	0.776	0.855	0.666
Perceived Behavioral Control	0.713	0.831	0.622
Subjective Norm	0.748	0.799	0.501
Attitude	0.753	0.822	0.537
Perceived Safety	0.777	0.807	0.516
Buying Impulse	0.771	0.804	0.508
Purchasing Intentions	0.927	0.911	0.719

In the attempt to analyze the acceptability of the created SEM, the model fit indices were accounted for after modification indices. For the following indices presented in Table 4, a 0.80 threshold was set by Gefen et al. [46] for the minimum acceptable value. In accordance, the root mean square error was set by Steiger [47] to be less than 0.70 for acceptable model output. From the results, all the parameters achieved the minimum threshold. With that, the final causal effect among the latent variables were recorded as seen in Table 5.

**Table 4:** Model fit.

Goodness of fit measures of SEM	Parameter Estimates	Minimum cut-off	Suggested by
Incremental Fit Index (IFI)	0.924	>0.80	Gefen et al. [46]
Tucker Lewis Index (TLI)	0.906	>0.80	Gefen et al. [46]
Comparative Fit Index (CFI)	0.923	>0.80	Gefen et al. [46]
Goodness of Fit Index (GFI)	0.875	>0.80	Gefen et al. [46]
Adjusted Goodness of Fit Index (AGFI)	0.840	>0.80	Gefen et al. [46]
Root Mean Square Error (RMSEA)	0.066	<0.07	Steiger [47]

**Table 5:** Direct, indirect, and total effects.

No	Variable	Direct Effect	P-Value	Indirect Effect	P-Value	Total Effect	P-Value
1	BI → PBC	0.307	0.019	–	–	0.307	0.019
2	BI → AT	0.742	0.010	–	–	0.742	0.010
2	BI → SN	0.564	0.021	–	–	0.564	0.021
3	BI → PI	0.556	0.012	0.234	0.044	0.790	0.015
4	SAF → PBC	0.144	0.022	–	–	0.144	0.022
5	SAF → AT	0.183	0.009	–	–	0.183	0.009
6	SAF → SN	0.170	0.016	–	–	0.170	0.016
7	SAF → PI	–	–	0.078	0.033	0.078	0.033
8	U → PR	0.894	0.010	–	–	0.894	0.010
9	U → PS	0.780	0.008	–	–	0.780	0.008
10	U → SE	0.748	0.020	–	–	0.748	0.020
11	U → PBC	–	–	0.644	0.007	0.644	0.007
12	U → AT	–	–	0.490	0.010	0.490	0.010
13	U → SN	–	–	0.348	0.005	0.348	0.005
14	U → PI	–	–	0.266	0.005	0.266	0.005
15	PR → PBC	−0.293	0.014	–	–	−0.293	0.014
16	PR → AT	0.203	0.025	–	–	0.203	0.025
17	PR → PI	–	–	−0.048	0.028	−0.048	0.028
18	PS → PBC	0.208	0.015	–	–	0.208	0.015
19	PS → AT	0.117	0.014	–	–	0.117	0.014
20	PR → PI	–	–	0.069	0.049	0.069	0.049
21	SE → PBC	0.994	0.007	–	–	0.994	0.007
22	SE → AT	0.290	0.035	–	–	0.290	0.035
23	SE → SN	0.465	0.006	–	–	0.465	0.006
24	SE → PI	–	–	0.340	0.025	0.340	0.025
25	PBC → PI	0.257	0.044	–	–	0.257	0.044
26	AT → PI	0.136	0.049	–	–	0.136	0.049
27	SN → PI	0.096	0.048	–	–	0.096	0.048

## 5 Discussion

With the evident increase of violence among women, whether at home, in the workplace, or outdoors, the need for purchasing intention behavior should be explored. The knowledge obtained from the findings of this study would help promote different types of safety tools among women. Several studies have either focused on an item as a safety tool, but no studies considered analysis why women would want to purchase these tools for safety reasons. For the complete analysis, the PMT aspect of the framework would cover the coping and threat appraisals while behavioral aspects were measured from the TPB framework. With the causal relationship, different relationships were obtained. This is primarily important to decipher because changing times causes changing motivation and buying behavior among consumers [48]. Presented in Table 6 are the summarized hypotheses output.

**Table 6:** Hypotheses summary.

Hypothesis No.	Relationship	Decision
1	U → PR	Accept
2	U → SE	Accept
3	U → PS	Accept
4	PR → PBC	Accept
5	PR → AT	Accept
6	PS → PBC	Accept
7	PS → AT	Accept
8	SE → PBC	Accept
9	SE → SN	Accept
10	SE → AT	Accept
11	PBC → PI	Accept
12	SN → PI	Accept
13	AT → PI	Accept
14	SAF → PBC	Accept
15	SAF → AT	Accept
16	SAF → PBC	Accept
17	SAF → PI	*Rejected*
18	BI → PBC	Accept
19	BI → AT	Accept
20	BI → PBC	Accept
21	BI → PI	Accept

Self-efficacy showed the highest significant effect on the TPB domains with an indirect effect on purchasing intentions. From which perceived behavioral control was seen to be greatly affected, followed by subjective norm, and attitude. The sequence of affected TPB domains is similar with the study of Liu et al. [33]. Their study showed that the sequence of behavioral aspects is affected by circumstances of how the product is being used. In line with this study, it was seen that women felt that they are easy target in the streets, but they can protect themselves. In that case, caring self-defense tool would help them, and that they have safety plans to deal with the possible risk. With the increased crime in the country, self-efficacy can be justified as the most influential factor. The protective behaviors of individuals, especially women, affects their coping mechanism behaviors [49]. To which, it was highlighted that the perception of environmental safety affects a great deal on how they would want to protect themselves. Since women are said to be prone to victimization, Strawinski and Celinska-Kopczynska [26] expounded on their ability to protect themselves. In this study, it was seen that more behavioral intentions are seen through purchasing safety tools. However, an indirect effect is seen – which means that women are more affected by people around them and by their own precaution to purchase safety tools.

Buying impulse showed the second highest relationship among TPB domains and affected purchasing intentions. From which, attitude was the most influential, followed by subjective norm, and then perceived behavioral control. The ability to have peace of mind upon purchase of safety tools with positive reviews promoted the behavioral relationship. Women respondents indicated that the price is not an issue, rather they are focused on its efficiency and have the impulse to purchase safety tools without second guessing. Kumar et al. [32] presented that psychological factors play a big role in purchasing intentions. Similarly, Rodrigues et al. [48] explained that stimuli for purchasing also is a determinant for purchasing behaviors. Since the increase of violence among women are evident worldwide, the impulse to self-protection greatly impacted and influenced women's protective behavior. As explained in the study of Iyer et al. [50], buying impulse are affected by other stimuli. In relation to this study, it is the awareness of women of the danger as victims of abuse and violence. The promotion of safety affects women protective behavior through buying impulse of purchasing of safety tools [51].

Under the PMT latent variables, understanding safety presented the highest contributing factor affecting the perceived risk, perceived severity, and self-efficacy. Evident from the earlier discussions, the indicators showed that women are aware of what is happening and that they understand safety concerns due to the imminent crimes and violence happening in the country. They know which areas are rather prone to danger, and they understood and are aware that surrounding people may be dangerous. Since violence may be prevalent in streets [2,3], workplaces [30], and even at home [9], women are more meticulous of the environment they are at. This therefore heightened their protective behavior due to the perception of risks and dangers, how severe something may happen, and thus have a positive intention for self-protection. As explained in the study of Author 2 et al. [17], the individuals who are knowledgeable and understands what about the case and aftermath affects more the three domains under coping and threat appraisal. There is also an indirect effect of understanding to the domains of TPB in this study, similar to that of Kurata et al. [18]. It was seen from their study that understanding how danger impacts an individual would lead to a high and positive relationship on intentions.

In addition, perceived severity affected perceived behavioral control and attitude. Women are knowledgeable of the severity of crimes and violence due to recent events, especially in streets. They do believe that women are more at risk than men, similar to the findings of Runyan et al. [10]. Moreover, wondering alone in the streets are seen to have severe aftermath when danger arise. Contrasting to the findings of Qui et al. [52], perceived severity was seen to be an insignificant latent variable in terms of people's intention. Their study focused on the emotional aspect, showing no significance on people's intention. With their topic focusing on pleasantry, enjoyment, and safe environment, evident perception of severe danger is not seen. Thus, it could be deduced that protective behavior through threat appraisals is more likely positive when unsafe environment is present [17]. With the prominent violence even at home, women are more likely taking the extra step for self-protection [3]. In relation to the demographic characteristics, most respondents are travelling back and forth workplaces or school which shows why heightened threat appraisals are evident from the items. There is a perception of purchasing self-defense tools for their own safety measure. Thus, an indirect effect on purchasing intention was seen from the results.

On the other hand, perceived risk was seen to have a negative direct effect on perceived behavioral control, positive on attitude, and indirect negative effect on purchasing intentions. Negative results are justified since women respondents showed that danger is evident when they are alone, not carrying weapon may be dangerous especially in the current time, and that the places they pass through are mostly prone to crimes. Recent studies such as that of Ferrer and Klein [53] and Palau-Saumell et al. [54] justified the negative impact of health-related risks on consumer behavior. It was seen that people would be more willing to protect oneself if risk is evident. In addition, socio-demographic status greatly affects the perception of risks [55]. Since Fontaneda et al. [27] expounded on the risk prevalence among women, the need to develop mitigation plans and protection among women should be emphasized. Though present protection plans, and helplines are present, perpetrators are still prominent as they would see women as easy targets. With that, perceived safety among women was seen to be affecting their behaviors.

For the perceived safety latent variable, close relationship was seen among the behavioral domains. Women believed that carrying safety tools would reduce their risks, are more confident travelling alone, can avoid street crimes, and avoid prominent areas that has high crime rates. Marshall [56] reiterated the knowledge on safety and practices have great effects on safety culture both in households and workplace. It could be deduced that when people would understand the safety concerns, a positive influence towards their behavior would be evident. In line with this study, perception of safety affected the domains of behavioral aspect among people; but a highlight that the perception of risk and severity, with positive self-efficacy for self-protection had higher significant effect. This is because recent crimes and violence among women are increasing, especially during the COVID-19 pandemic [57]. To which, perception of safety in this study affected the behavior in a sense that they will purchase safety tools for self-protection, but an interesting finding that it is not directly correlated. This indicates that women will purchase safety tools mainly for self-protection due to recent events and may not promote the purchasing behavior when safe environment is available. Therefore, an indirect effect on purchasing intention was seen.

Among the domains under TPB, perceived behavioral control was seen to be most distinct, followed by attitude, and subjective norm on purchasing intentions. They have the control to protect themselves as seen from the items considered. They have indicated that self-defense tools are beneficial and can increase safety as to why they have the intention to purchase. They also believe that buying safety tools are beneficial, they feel worried, anxious, and feels that buying safety tools are now a responsibility. Lastly, influence of others affected their decision of purchasing intentions since people around them think that they need to acquire safety tools for self-protection, there is also encouragement and other people they know already have safety tools with them. Contrasting to the findings of Kumar et al. [58] and Kumar and Nayak [32], their study focused on green consumer which showed that attitude, subjective norm, and perceived behavioral control were evident factors. On the note where people's well-being, health, and self-protection are at play [18], women's own control to safety are greatly considered. It is their own perception that affects their purchasing intention rather than influence from others. Highlights on the purchasing intentions showed that women are inclined to purchasing safety tools with recommendations and reviews on the types. They find it timely, relevant, and necessary to have safety tools. Moreover, the intention to purchase is likely due to the present crime rates that is happening in the country.

### 5.1 Practical implications and managerial insights

From the findings, it could be deduced that sellers may opt to advertise current events with their product and how it is used. Moreover, how their product may be considered for self-protection. From the findings, most women wanted to have the pepper spray, followed by stun guns/taser, and alarms. Low choices were seen with pocketknives, tactical knives and pens, and spikes. It could therefore be deduced that women would tend to have distance with perpetrators against crimes and violence. In relation to the latent variables and their indicators, the current perception for crimes is quite high – high risks and severity, which shows why they opted to consider self-protection that may help them escape from danger. The results showed that imminent risks are significant aspect as to why women would purchase self-defense tools. Therefore, marketing strategies involving these factors may be considered. The current scenarios should also be mitigated by government officials since the rise of violence is evident. Law enforcement, protection agencies, and help desks should always be available and should have ways to reduce risks felt by women in different countries. In addition, partnership with manufacturers of self-defense tools and the government may help women and provide insights of self-protection. This way, perpetrators would know and understand that this unlawful behavior are already closely monitored. The current generation and implementations are lacking the prevalent risk reduction of crimes and violence that is why perpetrators are still prominent. The findings of this study would provide a gateway as benchmark for how women feel, behave, and seeks protection. Implementing the suggestion with other aspects would end up promoting the overall safety of a country, not just for women, but for all victims against crime and violence.

### 5.2 Theoretical implications

For the perspective considering the integrated theories utilized in this study, it could be deduced that the protective behavior through the coping and threat appraisals, together with the behavioral domains could be utilized in any risk or health-related studies. With the integrated theories described in the literatures to be used in natural disasters, mitigation, and response [[16], [17], [18], [19]], as well as health [59]; this study presented that the integrated theory may be used in studies such as victimization and crimes. It could be seen that the PMT presents a better integration with behavioral theories such as TPB as it provides more complex and detailed aspect of behavior. In relation to other studies, minimal findings and limitations are evident when sole theories are being implemented. Based on this study's findings, a lot of applications can be used with the integrated PMT and TPB. Since the available literatures are commonly utilized in natural disasters and health-related human behavior studies, a great contribution on available literature is seen on the application of the applied extended TPB integrated with PMT. On addition, most studies have recommended the holistic measurement on the behavioral aspects [12], to which, this study was able to achieve and provide with the theoretical framework considered. With the extension done, it could also be considered and utilized in different aspect of studies that may be applied by different product evaluation and related studies about safety – even in different countries. In addition, it is suggested to then expand the measurement to consider mediating factors and other extended latent for more aspects to be analyzed.

### 5.3 Limitations and future research

Despite the positive results, several limitations were still considered with this study. First, only behavioral intentions incorporating safety perception were analyzed. It is suggested to consider other cognitive factors and structured interview to measure the weights of perceptions among women. Second, this study analyzed data coming from online survey – presenting results that may not be as diverse as in-person purposive sampling to separate urban and rural residences, age, and other demographic classification which may affect purchasing intentions. Third, only structural equation modeling was considered. Several studies have suggested the incorporation of machine learning ensemble to justify the nonlinear relationship that may present other sequence of significant relationship [19]. Moreover, clustering technique with the consideration of which type of safety tool they would want to consider based on demographic factors may provide more managerial insights and practical implications.

## 6 Conclusion

The rise of crime rates and violence both in household and outdoors are rising, especially in the period of the COVID-19 pandemic. The need for self-protection has been highlighted to be concerning with different studies pertaining to safety in public, even in workplaces. Women are seen to be more vulnerable and are targeted by perpetrators. Thus, the need to have self-protection through the purchase and obtainment of safety tools should be explored. This study was able to establish how perception of safety and vulnerability, together with the women's understanding to safety brought positive behavior towards intention to purchase safety tools. It could also be deduced that before threat arise, women are inclined to have threat appraisals due to available news, safety concerns, and rise of vulnerability from crimes and violence.

This study was able to present a comprehensive analysis on safety concern and protective behavior through the use of the extended TPB integrated with PMT. The model considered in this study could be posited as something that could be utilized to assess protective behavior among individuals in different fields of safety, and from related studies, natural disasters, calamities, and health. As a contribution, this study provided basis for the use of the integrated theories as a way to assess protective behaviors. Perception of risks and severity of crimes and violence played a significant role in the positive intention for purchasing safety tools. Understanding and knowledge based on current events presented the urge of women to acquire safety tools. This, therefore, should be considered by marketers to promote selling their products. It is suggested as well that the government promote the use of these safety tools – partnering with marketing and developers of the products to suggest protection of people in the country.

The current generation and implementations are lacking the prevalent risk reduction of crimes and violence that is why perpetrators are still prominent. The findings of this study would provide a gateway as benchmark for how women feel, behave, and seeks protection. Implementing the suggestion with other aspects would end up promoting the overall safety of a country, not just for women, but for all victims against crime and violence. Lastly, it is suggested to expand the measurement to consider mediating factors and other extended latent for more aspects to be analyzed.

## Author contribution statement

Ardvin Kester S. Ong; Tyrone Wyeth O. Arceno; Allyza R. Padagdag; Wayne Ralph Lee B. Saragat; Hershey Reina Mae S. Zuñiga; Ma. Janice J. Gumasing: Conceived and designed the experiments; Performed the experiments; Analyzed and interpreted the data; Contributed reagents, materials, analysis tools or data; Wrote the paper.

## Data availability statement

Data will be made available on request.

## Declaration of competing interest

The authors declare that they have no known competing financial interests or personal relationships that could have appeared to influence the work reported in this paper.
