# Supplementary material for: Antecedents of behavioral intentions for purchasing safety tools among women
Source: Heliyon. 2023 Jul 5;9(7):e17994. doi: 10.1016/j.heliyon.2023.e17994 (PMC10395343; doi:10.1016/j.heliyon.2023.e17994)
Supplement: Multimedia component 1 [file mmc1.docx]

| **Appendix.** Questionnaire Items | | |
| --- | --- | --- |
| **Latent variable** | **Code** | **Construct** |
| Understanding Safety | **U1** | I aware of the different crimes timely in my recent locations. |
|  | **U2** | I understand the dangers of wandering alone in my location. |
|  | **U3** | I am aware of locations prone to street danger. |
|  | **U4** | I am aware that the people around me may be dangerous. |
| Perceived Risk | **PR1** | I believe that not carrying a safety weapon can increase my risk to crimes. |
|  | **PR2** | I think that I am at risk of danger while alone in the streets. |
|  | **PR3** | Based on recent events, venues of my frequent visits may be prone to crimes. |
|  | **PR4** | The risks are higher if i go to places that i dont recognize. |
| Self-Efficacy | **SE1** | I believe that I can be an easy target in street crimes. |
|  | **SE2** | I believe that i can properly protect myself from danger. |
|  | **SE3** | I believe that bringing self-defense tools can make me safer. |
|  | **SE4** | I have a safety plan on how to deal with street crimes. |
| Perceived Severity | **PS1** | I believe that recent crimes can result to great danger and even death. |
|  | **PS2** | I believe that recent crime happenings are a severe threat to one's safety in the streets. |
|  | **PS3** | I believe that women are more in risk to street dangers than men. |
|  | **PS4** | I find that street crime hinders my safety wandering alone outside. |
| Perceived Behavioral Control | **PBC1** | I believe that carrying a self-defense tool is a benefit. |
|  | **PBC2** | I know how to search risks that are timely in my frequent locations. |
|  | **PBC3** | I believe that buying a self-defense tool is entirely up to me. |
|  | **PBC4** | I believe I can increase safety measures if i have a self-defense tool. |
| Subjective Norm | **SN1** | I feel responsible to carry self-defense tools in order to protect myself. |
|  | **SN2** | Most of the people I know believe that carrying tools is helpful to protect oneself. |
|  | **SN3** | My family encourage me to carry a self-defense tool. |
|  | **SN4** | My female friends always carry a self-defense tool. |
| Attitude | **A1** | I think that buying self-defense tools is worth it. |
|  | **A2** | I worry about the effects of not carrying a self-defense tool. |
|  | **A3** | I feel anxious whenever I hear news about threats in the streets. |
|  | **A4** | I think that buying self-defense tools is a responsibility. |
| Perceived Safety | **SAF1** | I believe that my risk in street crimes will lower when carrying a self-defense tool. |
|  | **SAF2** | I feel confident to wander alone in the street with a weapon. |
|  | **SAF3** | I think that I can completely avoid street crimes with a self-defense tool. |
|  | **SAF4** | I believe that if I avoid suspicious alleyways I will be safe. |
| Purchase Intention | **PI1** | I intent to buy self-defense tools. |
|  | **PI2** | I intent to seek buyer reviews/experience about self-defense tools. |
|  | **PI3** | I am more likely to buy self-defense tools because they are timely essentials. |
|  | **PI4** | I am more likely to purchase self-defense tools due to the current events. |
| Buying Impulse | **BI1** | Buying a self-defense tool will benefit my peace of mind. |
|  | **BI2** | I do not mind the price of self-defense tools. |
|  | **BI3** | The efficiency of the self-defense tools is critical for me. |
|  | **BI4** | I suddenly want to purchase self-defense tools that I didn't plan on purchasing. |
